# Evaluation of Immunotoxicity Induced by Organophosphorus Pesticide Malathion

**DOI:** 10.3390/toxics14040279

**Published:** 2026-03-26

**Authors:** Weichunbai Zhang, Minhan Lou, Ling Yong, Xiao Xiao, Chunlai Liang, Wei Wang, Hui Yang, Xudong Jia, Yin Wang, Yan Song

**Affiliations:** 1 NHC Key Laboratory of Food Safety Risk Assessment, China National Center for Food Safety Risk Assessment, Beijing 100022, China; 2 School of Food Science and Engineering, Hangzhou Medical College, Hangzhou 310012, China; 3 School of Public Health, Southern Medical University, Guangzhou 510515, China

**Keywords:** malathion, immunotoxicity, immunosuppression, humoral immunity, lowest-observed-adverse-effect level

## Abstract

Malathion (MLT) is an organophosphate pesticide widely used worldwide. Due to its environmental persistence and accumulation in living organisms, concerns have been raised regarding its potential health effects beyond the classical mechanism of cholinergic inhibition, particularly its impact on immune function. In this study, we aimed to systematically evaluate the immunotoxicity of MLT in mice and identify the lowest-observed-adverse-effect level (LOAEL) for immunotoxic effects. Key parameters assessed included body and organ weights, hematological and clinical chemistry profiles, histopathological changes, and immune function indicators. The results showed that exposure to MLT, particularly at low and intermediate doses, led to a significant increase in thymus weight, along with marked reductions in interleukin-10 (IL-10) levels, neutrophils, polychromatic erythroblasts, and monocyte lineage cells. Histological examination revealed atrophy of splenic white pulp, indicating immunopathological alterations predominantly at these dose levels. In contrast, immunoglobulin G (IgG) levels increased in a dose-dependent manner, possibly reflecting a compensatory humoral response to the observed suppression of cellular immune components. Meanwhile, the plaque-forming cell (PFC) response exhibited a dose-dependent trend but was significantly inhibited only at the highest dose, suggesting a complex, non-linear effect on humoral immunity. Based on significant alterations in thymus weight, cellular immune parameters, and splenic histopathology observed at the lowest dose tested (16 mg/kg bw), this value was preliminarily identified as the LOAEL for MLT-induced immunotoxicity in mice.

## 1. Introduction

Malathion (MLT) has been widely used in agriculture and household pest control for years, and it is sometimes used to treat head lice on humans as well [1,2]. Since the long-term use of MLT began, it has been distributed in the environment, although ultraviolet light [3] and some microorganisms in the soils [4] continuously degrade it. Their exposure pathways were diverse, including occupational exposure, environmental media (such as air and water), and consumption of residual contaminated food. In some cases, occupational workers may have been exposed to MLT through inhalation or dermal contact when they did not wear protective gear during spraying activities. Except for occupational exposure, evidence suggested that the use of MLT for household pest control and the ingestion of water and food containing MLT increase human health risks, which were the primary exposure pathways for the general population [5]. MLT can persist in various crops, such as grains, fruits, and vegetables, and enter the human body through long-term, low-dose dietary intake, posing potential chronic health risks [6,7]. Based on previous studies, MLT was linked with various diseases, including allergic contact urticaria [8], depression [9], hypothyroidism [10], and prostate cancer [11,12], and the International Agency for Research on Cancer classified it as “probably carcinogenic to humans” (Group 2A) [13]. Experimental evidence showed that this commonly used pesticide had the potential for neurotoxicity [14], developmental toxicity [15], genotoxicity [16], and immunotoxicity [17], and these findings were particularly important for pesticide safety management. Therefore, a comprehensive assessment of its potential toxic effects is of great significance for safeguarding public health.

In addition to its recognized neurotoxicity, the immunotoxicity of pesticides has increasingly become a focus of toxicology research and risk assessment. Acting as a defensive barrier for the body, the immune system is highly sensitive to xenobiotics; it has been well documented that adverse effects on the immune system often occur at doses lower than those causing general toxicity [18,19]. This heightened sensitivity underscores the importance of evaluating immunological endpoints in safety assessments. Therefore, it was particularly important to focus on the study of pesticide immunotoxicity and to develop a comprehensive, scientific, and specific immunotoxicity testing guideline for safety assessment and management. Given the profound impact that immunotoxicity may have on public health, the United States has set higher requirements for pesticide registration management. In addition to acute, subchronic, chronic, reproductive, developmental, and genetic toxicity, and metabolic studies of pesticides, the immunotoxicity of pesticides is also required for registration. The United States Environment Protection Agency (USEPA) has made several revisions to its previous guidelines for evaluating the immunotoxicity of pesticides, highlighting the necessity and regulatory importance of systematic assessment of pesticide immunotoxicity [20,21,22]. Notably, studies have demonstrated that even low-dose pesticide exposure can induce immune responses and alter immune-related gene expression [23], further supporting the need for comprehensive immunotoxicity evaluation. Therefore, it was particularly important to study the immunotoxicity of pesticides, including MLT, to develop comprehensive, scientific, and specific immunotoxicity thresholds for safety assessment and management.

While MLT has a long history of use, comprehensive assessments of its immunotoxicity in accordance with current regulatory guidelines have not yet been adequately conducted. To date, no clear immunotoxicity threshold has been established for MLT, and comprehensive dose–response studies assessing multiple immune function endpoints are scarce [24]. Existing research has largely focused on high-dose acute exposure or the detection of single immune parameters [24], which may not adequately reflect the potential health risks associated with chronic low-dose exposure scenarios typical of real-world settings. Therefore, a systematic evaluation incorporating a range of immunological endpoints across multiple dose levels was needed to characterize the immunotoxic potential of MLT and to inform evidence-based safety thresholds.

Although the overall novelty of immunotoxicity research on organophosphates may be moderate, the present study addressed key gaps by providing the first systematic evaluation of MLT immunotoxicity in accordance with current USEPA testing guidelines. Unlike previous studies that examined isolated endpoints or high-dose effects, this study incorporated a multi-dose design encompassing a comprehensive panel of immunological parameters, including organ weight, hematological profiles, histopathological examination, and functional immune assays. This approach enabled the identification of sensitive immunological endpoints, thereby facilitating the determination of a preliminary immunotoxicity threshold for MLT. The findings were expected to provide a critical experimental basis for more accurate health risk assessment and informed regulatory decision making regarding MLT exposure.

## 2. Materials and Methods

### 2.1. Materials

MLT (purity 95%) was purchased from Hebei Hengshui Beifang Pesticide Chemical Co., Ltd. (Hengshui, China). Cyclophosphamide (CY) was purchased from Jiangsu Hengrui Pharmaceutical Co., Ltd. (Lianyungang, China). Sheep red blood cell (SRBC) was obtained from Beijing Laboratory Biology Technology Co., Ltd. (Beijing, China). FITC hamster anti-mouse CD3e, APC rat anti-mouse CD4, APC rat anti-mouse CD19, PE rat anti-mouse CD8a, PE rat anti-mouse CD49b, lysing buffer, and mouse CBA flex sets (interleukin-2 [IL-2], interleukin-4 [IL-4], interleukin-5 [IL-5], interleukin-10 [IL-10], interferon-γ [IFN-γ], tumor necrosis factor [TNF]) were obtained from Becton, Dickinson and Company (Franklin Lakes, NJ, USA). Mouse immunoglobulin G (IgG), immunoglobulin A (IgA), and immunoglobulin M (IgM) enzyme-linked immunosorbent assay (ELISA) kits were purchased from GenWay Biotech, Inc. (San Diego, CA, USA). Concanavalin A (ConA) and lipopolysaccharide (LPS) were obtained from Sigma-Aldrich Co. LLC (St. Louis, MO, USA). Roswell Park Memorial Institute (RPMI) 1640 medium was obtained from Thermo Fisher Scientific Inc. (Logan, UT, USA).

### 2.2. Animals

In this study, 6–8-week-old female BALB/c mice (SPF grade, body weight 18–22 g) were used. Animals were purchased from Beijing HFK Bioscience Co., Ltd. (Beijing, China) and maintained in a temperature-controlled (20–25 °C), relative humidity-controlled (40–70%), artificially illuminated, light/dark-cycle-controlled (10/14 h), and air-exchange-controlled (10–15 times/h) environment. The mice were fed a chow diet (Beijing HFK Bioscience Co., Ltd., Beijing, China, No. SCXK 2009-0008). All animals were observed within 3 days of arrival (experimental treatment prior to days 1–3). Nutritional propagation diets and water were available ad libitum during the 30-day feeding studies. The diets and water have been tested, and they did not contain MLT. At the end of treatment, mice were humanely euthanized. The study was approved by the Animal Experimental Welfare & Ethical Inspection Committee, China National Center for Food Safety Risk Assessment (No. 2012012).

### 2.3. Experimental Design

Before experimental treatments, animals underwent a 6-day acclimatization period. After 3 days of adaptive feeding (experimental treatment prior to days 4–6), mice were randomly assigned to groups of 10, each based on body weight. Specifically, all mice were first weighed and ranked by body weight. A random number table was then used to assign individuals to either the negative control group, three dosage groups, or the positive control group, ensuring a balanced distribution of body weights across all groups. In this study, a negative control group, three dosage groups, and a positive control group were established. The acute oral toxicity of MLT in BALB/c mice was determined using Horn’s method, yielding a median lethal dose (LD_50_) of 1030 mg/kg body weight. The highest MLT dose in the study was primarily based on the results of the oral acute toxicity study in mice (1/4 LD_50_). The dosages for the low-, middle-, and high-dose groups were 16 mg/kg bw, 65 mg/kg bw, and 258 mg/kg bw of MLT, respectively. These doses were selected to span from a no-observed-adverse-effect level (NOAEL) to a clearly toxic level based on acute toxicity data, thereby enabling determination of a lowest-observed-adverse-effect level (LOAEL) for immunotoxic effects. The low dose (16 mg/kg bw) was expected to produce no or minimal immunotoxicity, the middle dose (65 mg/kg bw) was chosen to elicit moderate effects potentially, and the high dose (258 mg/kg bw, approximately 1/4 LD_50_) was selected to assess immunotoxic responses below the threshold for overt acute toxicity, consistent with regulatory guidelines for immunotoxicity studies [20]. Both the negative control group and the positive control group of mice were gavaged daily with corn oil, and the mice in the positive control group were intraperitoneally injected with 200 mg/kg bw of CY 24 h before the end of the study. As a powerful immunosuppressant, CY was commonly used to establish an immunosuppressive animal model in immunotoxicology studies of food additives or chemicals [25]. According to a previous study in 2013, a single dose of 200 mg/kg BW of CY via intraperitoneal injection 24 h before the end of the study was recommended, as it effectively reduced immune function in animals [26]. This specific dose was selected based on the established literature demonstrating that 200 mg/kg CY induced significant and reproducible immunosuppression in mice without causing excessive mortality or overt toxicity, making it suitable as a positive control for evaluating immunotoxic effects in accordance with standard immunotoxicology study guidelines [25,26]. During the configuration process, complete dissolution was confirmed by visual inspection, ensuring a clear, homogeneous corn oil with no visible particulates. Housed the animals by group with corresponding doses of gavage during the study.

Mice were euthanized on day 30, and spleens were removed under sterile conditions. The mice had been immunized on day 25 by intraperitoneal injection with 0.2 mL of 2% (*v*/*v*) SRBC suspension in sterile saline, and the spleens were transferred to petri dishes containing 2 mL of HBSS. Finely ground the spleens and transferred the cell suspensions into tubes. Centrifuged for 10 min at 1000 rpm and washed twice in HBSS at 4 °C. Splenocytes were diluted to 5 × 10^6^ cells/mL in the culture medium (RPMI) for subsequent use.

### 2.4. Body Weight and Feeding Quantity

All animals were weighed at weekly intervals, and the gavage dose was adjusted according to the mice’s body weight over time. At the end of the experiment, the mice’s weights were recorded, and they were humanely euthanized.

### 2.5. Hematology and Clinical Chemistry Parameters

For hematological analysis, whole blood was collected from the retro-orbital plexus and transferred into tubes containing ethylenediaminetetraacetic acid dipotassium (EDTA-K2) as an anticoagulant. A COULTER Ac.T diff2 Hematology Analyzer (Beckman Coulter Corporation, Brea, CA, USA) was used to measure the following parameters: total leukocyte count (WBC), red blood cell count (RBC), hematocrit (HCT), hemoglobin (HGB), mean corpuscular hemoglobin (MCH), mean corpuscular volume (MCV), mean corpuscular hemoglobin concentration (MCHC), platelet count (PLT), red blood cell distribution width (RDW), prothrombin consumption test (PCT), mean platelet volume (MPV), and platelet distribution width (PDW), and the count and percentage of monocytes (MON), lymphocyte (LYM), neutrophils (NEUT), basophils (BAS), and eosinophils (EOS).

Blood for clinical chemistry analysis was collected as stated, without an anticoagulant. Spun the blood sample to obtain serum (4000 rpm/min, 10 min) in the Low Speed Centrifuge Model(Beijing Medical Centrifuge Factory, Beijing, China) and tested the clinical parameters with an automatic clinical analyzer (Hitachi 7080, Hitachi High-Technologies Corporation, Tokyo, Japan): aspartate aminotransferase (AST), alanine aminotransferase (ALT), albumin (ALB), total protein (TP), albumin to globulin (A/G), glucose (GLU), alkaline phosphatase (ALP), creatinine (CRE), urea nitrogen (BUN), cholesterol (CHO), triglyceride (TG), calcium (CA), sodium (NA), and potassium (K).

### 2.6. Peripheral Blood Lymphocytes Phenotyping

Collected whole blood in the same way and transferred it into tubes with anticoagulant (EDTA-K^2^) inside. Two kinds of three-color combinations of antibodies (antibodies of CD3e, CD19, and CD49b or antibodies of CD3e, CD4, and CD8) were used to stain 50 μL of blood cell suspension, respectively, for 20 min at room temperature in the dark. Added into 2 mL of RBC lysing buffer, spun, and kept for 20 min at room temperature in the dark. Centrifuged twice (1200 rpm min^−1^, 5 min), samples spun in 0.5 mL of PBS and analyzed on a FACSCalibur flow cytometer using CellQuest software (Pro 5.2.1, Becton, Dickinson, and Company, Franklin Lakes, NJ, USA). Appropriate isotype controls were used for compensation controls.

### 2.7. Bone Marrow Cell Classification

Bone marrow cells were collected from the left femurs of mice and prepared as smears. The percentages of promyelocytes, myelocytes, metamyelocytes, stab granulocytes, segmented granulocytes, basophilic normoblasts, polychromatic normoblasts, orthochromatic normoblasts, lymphocytic series, monocytic series, and others were determined.

### 2.8. Splenocyte Count

Removed the spleens under aseptic conditions and placed them in culture dishes containing Hank’s solution. Ground the spleens and transferred the cell suspensions into tubes. Centrifuged the cell suspensions for 10 min at 1000 rpm and washed twice in HBSS at 4 °C. Then, the splenocytes were suspended in 1 mL of complete culture medium to determine the splenocyte count.

### 2.9. Pathology

Animals were euthanized and dissected at the end of the experiment. Liver, kidney, spleen, thymus, lymph glands (axillary lymph nodes, cervical lymph nodes, mesenteric lymph nodes), Peyer’s patches, and bone marrow were removed. Weighed the organs and calculated the relative weights, and counted the number of Peyer’s patches on the small intestine. Fixed the organs in 10% neutral buffered formalin, processed for paraffin embedding, sectioned at 5 μm, made into slices with HE staining, and observed under the optical microscope, and all histological measurements were quantified using an image analysis system. Specifically, digital images were captured at 100× magnification, and the areas of splenic periarterial lymphatic sheaths, splenic germinal centers, lymph follicles in Peyer’s patches, and germinal centers in Peyer’s patches were manually outlined and automatically calculated by the software using pixel calibration.

### 2.10. Humoral Immunity Functions

#### 2.10.1. Plaque-Forming Cell (PFC) Assay

Mice were immunized on day 25 with 0.2 mL of a 2% (*v*/*v*) SRBC suspension in sterile saline by intraperitoneal injection. Five days later, the splenocyte suspensions were prepared as stated. Transferred 25 μL of splenocyte suspensions in RPMI 1640 medium (5 × 106 cells mL^−1^), which were supplemented with 10% fetal bovine serum and 1% penicillin-streptomycin solution, to a glass tube containing 50 μL of 10% (*v*/*v*) SRBC in SA buffer solution and 0.5 mL of agar solution (0.5 g mL^−1^ in HBSS, pH 7.2–7.4), and poured onto slides. The slides were inverted on a special frame after the mixtures were solidified and incubated (37 °C, 1.5 h). Added guinea pig sera to the slot between the glass slides and the bottom of the frame. Incubated for another 1.5 h (37 °C); then, plaque production was counted, and the results were expressed as the number of PFC/106 splenocytes.

#### 2.10.2. Serum Immunoglobulin Quantification

ELISA kits (Gen Way Biotech, Inc., San Diego, CA, USA) were used to determine the total IgA, IgG, and IgM levels in serum. The test steps were completely in accordance with the instructions. Mice were immunized as stated. One-hundred μL of blank, standard, or diluted serum samples were respectively added into the 96-well plates in duplicate, incubated (room temperature, 1 h), and washed 4 times with wash solution. Added enzyme–antibody conjugate (100 μL well^−1^), incubated (room temperature, 30 min), and washed 4 times with wash solution. Added TMB substrate solution (100 μL well^−1^), incubated (room temperature, 10 min), and added stop solution (100 μL well^−1^) to prevent further reactions. Finally, the absorbance (450 nm) of the contents in each well was determined within 15 min, using an ELISA Reader (BioTek, Winooski, VT, USA).

#### 2.10.3. Hemolysis Test

Collected whole blood and obtained the serum as stated. One mL of SA buffer solution, 0.5 mL of 10% (*v*/*v*) SRBC, 1 mL diluted guinea pig complement (1:8 diluted with SA buffer solution), and 2 μL mouse sera were mixed. Set the control tube without mouse serum. Kept the tubes in a water bath (at 37 °C, 15 to 30 min) and put them in an ice bath immediately to stop the reaction. Centrifuged (2000 r min^−1^, 10 min), collected 1 mL of supernatant, and added to 3 mL Drabkin solution (1.0 g of NaHCO_3_, 0.05 g of KCN, and 0.2 g of K_3_Fe(CN)_6_ dissolved in 1000 mL of still water). Meanwhile, 0.25 mL of 10% (*v*/*v*) SRBC and 3.75 mL Drabkin solution were thoroughly mixed and placed for 10 min as the positive control. Determined the absorbance (540 nm) and obtained the HC_50_ value by the formula ${\mathrm{HC}}_{50}=\left({\mathrm{OD}}_{1}/{\mathrm{OD}}_{2}\right)\;\times \;500\;$(OD_1_ = the OD value of the sample well minus that of the control well, OD_2_ = the OD value of the positive control well minus that of the control well).

#### 2.10.4. Serum Cytokine Quantification

Collected whole blood without anticoagulant, obtained the serum as stated, and assayed for the levels of cytokines by Mouse CBA Flex sets, including IL-2, IL-4, IL-5, IL-10, IFN-γ, and TNF. The assay fully enforced the descriptions in the manufacturer’s instructions. Transferred 50 μL of mixed capture beads into the tubes, added 50 μL of standard, blank, or serum samples to the appropriate tubes, added PE detection reagent into each tube, and incubated in the dark (room temperature, 2 h). After incubation, the samples were washed and spun in 300 μL of wash buffer. Analyzed on a FACSCalibur flow cytometer with CellQuest software (Pro 5.2.1).

### 2.11. Cellular Immunity Functions

#### 2.11.1. Mitogen-Induced Splenic Lymphocyte Proliferation

ConA and LPS were dissolved in distilled water (100 μg mL^−1^), respectively. The splenocyte suspensions (3 × 10^6^ cells mL^−1^) were prepared as stated, and 1 mL of the suspension was added to the 24-well plate for culture with 75 μL of the mitogen solutions (at 37 °C, 5% CO_2_, for 68 h). In the control well, 75 μL of distilled water was added to replace the mitogen. Four h before the end of incubation, discarded 0.7 mL of the supernatant from each well, immediately added 0.7 mL of RPMI 1640, added 50 μL/well of freshly prepared MTT solution (5 mg mL^−1^, dissolved in PBS, pH 7.2), and continued incubation. Four h later, 1 mL/well of acid-isopropanol (4 mL of 1 mol L-1 HCl added to 96 mL of isopropanol) was added and mixed to ensure the purple crystals were dissolved. Transferred the solution to 96-well plates in triplicate and determined the absorbance (570 nm) of the contents in each well. The calculation results were the OD value of the sample well minus the OD value of the control well.

#### 2.11.2. Delayed-Type Hypersensitivity (DTH)

Mice were immunized as stated. On day 30, the thickness of the left rear footpads of the mice was determined with a Vernier caliper (Changchun, China). Hypodermically injected the left rear footpads with 20 μL of 20% (*v*/*v*) SRBC and measured the thickness 24 h later. The extent of DTH was expressed by the difference in footpad thickness before and after hypodermic injection.

#### 2.11.3. Cytotoxic T-Lymphocyte (CTL) Assay

Splenocyte suspensions were prepared (6 × 10^7^ cells/mL) as the effector cells. Added 0.5 mL of splenocyte suspensions and an equal amount of target cells, P815 cell suspensions (1.2 × 10^6^ cells/mL), into the 24-well round-bottom plate and incubated for 5 days at 37 °C with 5% CO_2_. Collected the cultures, washed in Hank’s solution, and resuspended the cells in RPMI 1640 medium (2 × 10^7^ cells mL^−1^). Added 100 μL of effector cells (2 × 10^7^ cells mL^−1^) to triplicate wells in round-bottom plates and added 100 μL of target cells (2 × 10^5^ cells mL^−1^) to each well for a total of 200 μL/well. Spontaneous LDH release and total LDH release of target cells were determined by adding culture solution, 2.5% Triton solution, and effector cell suspension, respectively, as well as the effector cell control. All plates were incubated for 6 h (37 °C with 5% CO_2_) and spun (1000 r min^−1^, 5 min). Transferred 100 μL well^−1^ of supernatant to another 96-well round-bottom plate, added 50 μL of freshly prepared LDH substrate solution in each well, and incubated at room temperature for 30 min in the dark. Fifty μL per well of 1 mol L^−1^ HCl solution was added to stop the reaction. The absorbance (492 nm) was determined. The results were calculated by the equation $\%\;\mathrm{CTL}\;\mathrm{cell}\;\mathrm{activity}=\left[\left(E\;-\;S\;-\;C\right)/\left(M\;-\;S\right)\right]\times 100$ (where E = experimental release of effector/target co-culture; S = spontaneous target cell LDH release; C = release of effector cell control; M = total target cell LDH release).

### 2.12. Non-Specific Immunity

#### 2.12.1. NK Cell Activity Assay

One-hundred μL of splenocyte suspensions (2 × 10^7^ cells mL^−1^) was prepared as stated and plated in a 96-well round-bottom plate as effector cells. One-hundred μL of YAC-1 cell suspensions (4 × 10^5^ cells mL^−1^) was prepared and added as target cells. Added 100 μL of culture solution and 2.5% Triton solution, respectively, to determine the spontaneous LDH release and the total LDH release of target cells. All tests were performed in triplicate. The plates were incubated for 4 h at 37 °C with 50% CO_2_ and spun for 5 min at 1500 r min^−1^. Then, 100 μL well^−1^ of supernatant was removed to another 96-well flat-plate, incubated with 100 μL of freshly prepared LDH substrate solution (0.05 mol L^−1^ lithium lactate, 6.6 × 10^−4^ mol L^−1^ 2p-iodophenyl-3p-nitrophenyl tetrazolium chloride, 2.8 × 10^−4^ mol L^−1^ phenazine metosulphate, and 1.3 × 10^−3^ mol L^−1^ nicotinamide nucleotide NAD in 0.2 mol L^−1^ Tris-HCl buffer, pH 8.2) at room temperature in the dark for 10 min. Stopped the reaction by adding 30 μL well-1 of HCL (1 mol L^−1^). Determined the absorbance (490 nm) and calculated the results with the equation %$\;\mathrm{NK}\;\mathrm{cell}\;\mathrm{activity}=\left[\left(E\;-\;S\right)/\left(M\;-\;S\right)\right]\;\times \;100$ (where E = experimental release of effector/target co-culture; S = spontaneous target cell LDH release; M = total target cell LDH release).

#### 2.12.2. The Carbon-Clearance Test

The macrophage activity was assessed using a carbon-clearance test. On day 30, mice were injected with diluted ink (10 mg/mL) via the caudal vein. At 2 min or 10 min after the injection, 10 μL of the blood was collected as stated and added to 2 mL of 1 mg/mL Na_2_CO_3_ solution. Then, the absorbance (600 nm) of the mixed samples was measured and the index of phagocytosis was calculated by the formula $\mathrm{Index}\;\mathrm{of}\;\mathrm{phagocytosis}\;=\;\left[\mathrm{BW}/\left(\mathrm{LW}\;+\;\mathrm{SW}\right)\right]\;\times \;\left[\left(\mathrm{lg}{\mathrm{OD}}_{1}\;-\;\mathrm{lg}{\mathrm{OD}}_{2}\right)/\left(t_{2}\;-\;t_{1}\right)\right]1/3$ (BW: body weight, LW: liver weight, SW: spleen weight, OD1: the absorbance (600 nm) 2 min after the injection of ink, OD_2_: the absorbance (600 nm) 10 min after the injection of ink).

### 2.13. Statistical Analysis

This study was conducted as a single experiment, with each mouse serving as an individual biological replicate. All data were derived from independent animals. All data were analyzed using SPSS software (version 17.0). The values were presented as means ± SD. Comparisons between multiple groups were carried out using one-way ANOVA followed by Bonferroni post hoc comparison tests when equal variances were assumed and Dunnett’s T3 post hoc tests when the equal variances assumption was not met. *p* < 0.05 was considered a statistically significant difference.

## 3. Results

### 3.1. Effects on Body and Organ Weights

As seen in Figure S1, there was no significant difference in periodic body weight among the groups in all weeks (Table S1). The relative weight of the kidney and the absolute and relative weights of the liver in the high-dose group were significantly greater than those in the negative control group, the low-dose group, and the middle-dose group; the relative and absolute weights of thymus in the low-dose group and the middle-dose group were significantly higher than those in the negative control group (Table S2).

### 3.2. Effects on Hematology and Clinical Chemistry Parameters

**Figure 1:**
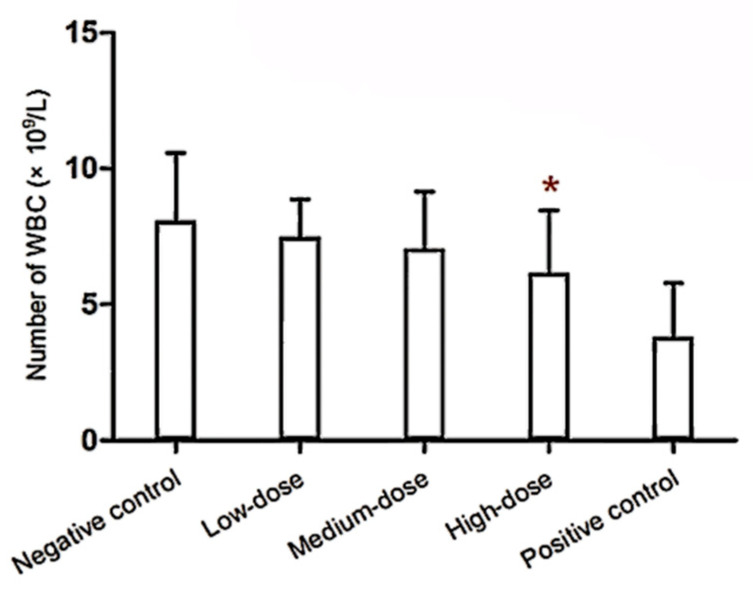
Effect on the number of WBC in BALB/c mice. Note: * statistically significant difference from the negative control group and the positive control group at *p* < 0.05. The number of WBCs decreased with increasing MLT concentration, and the WBC level in the high-dose group was significantly lower than that in the negative control group.

The high dose of MLT caused a significant decrease in the WBC count compared with the negative control group and showed a linear dose–response relationship (Figure 1). Although the PLT count in the high-dose group was significantly lower than in the low-dose group, it did not differ significantly from the negative control group. The NEUT levels in the low-dose and middle-dose groups were significantly lower than those in the negative control group. Moreover, the count and percentage of BAS in the medium-dose group were higher than those of the negative control group (Table 1).

There were no significant differences in ALT levels between the treated groups and the negative control group; however, ALT levels in the high-dose group were significantly higher than those in the low-dose and the middle-dose groups. The serum TP level in the high-dose group was significantly lower than that in the negative control group, and the serum TP levels in the middle- and high-dose groups were significantly lower than those in the low-dose group. The ALB levels in the high-dose group were significantly lower than those in the negative control, low-dose, and medium-dose groups, and there was a significant dose–response relationship among the three dosage groups. Although the GLU level in the middle-dose group was significantly higher than in the negative control and high-dose groups, there was no dose–response relationship among the groups. The blood glucose indices were within the normal range of laboratory history, so they had no biological significance. The serum CHO level in the high-dose group was higher than in the negative control and low-dose groups (Table 2).

### 3.3. Effects on Phenotypic Analysis of Peripheral Blood Lymphocytes

Compared with the negative control group, the percentage of B lymphocytes (CD^3−^CD^19+^) showed a dose-dependent increasing trend with MLT treatment. Specifically, the B-cell percentages in the middle-dose (36.1% ± 9.2%) and high-dose (37.0% ± 7.3%) groups were significantly higher than those in the negative control group (29.2% ± 4.7%). From a biological perspective, the dose-dependent increase in B lymphocytes at middle and high doses may indicate immune stimulation or a compensatory proliferative response to MLT exposure. This pattern could reflect enhanced humoral immune activity, consistent with reports that organophosphorus compounds can modulate B-cell function at subtoxic doses. Regarding T lymphocytes (CD^3+^CD^8+^), the percentage was significantly elevated only in the middle-dose group compared with the negative control, whereas the low- and high-dose groups showed no significant differences. This isolated increase at the middle dose, without a corresponding rise at the high dose, indicates the absence of a clear dose–response relationship for this subset. The lack of dose dependence suggested that the increase in T lymphocytes may represent a transient or non-linear immunomodulatory event rather than a direct toxic effect, possibly involving compensatory regulatory mechanisms at intermediate exposure levels. Other lymphocyte subsets were presented in Table S3 and Figure S2.

### 3.4. Effects on the Number of Splenocytes and Bone Marrow Cells

There was no significant difference in the number of splenocytes (Table 3). The number of polychromatic normoblasts and monocyte series in the medium-dose group was statistically lower than that in the negative control group (Table S4). Although these changes were limited to specific cell lineages and did not affect overall splenocyte counts, they may represent early indicators of hematopoietic toxicity. Bone marrow is a primary site of hematopoiesis, and alterations in erythroid precursors (polychromatic erythroblasts) and the monocyte series could reflect subtle disruptions in myeloid differentiation or in the function of the bone marrow microenvironment following MLT exposure. The fact that these effects were observed only at the middle dose, without a clear dose–response pattern, suggests a possible non-monotonic response or a threshold effect that warrants further investigation. These findings, while preliminary, highlighted the importance of examining bone marrow cellular composition as a sensitive endpoint in immunotoxicological assessments, as changes may precede overt peripheral blood alterations.

### 3.5. Histopathological Examination

**Figure 2:**
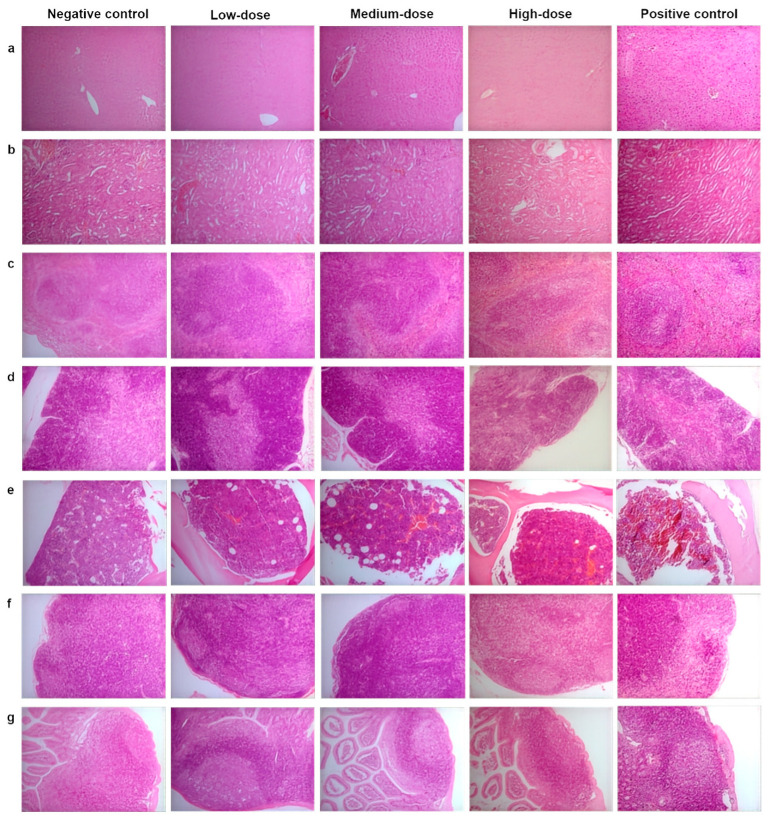
**Histopathology of various tissues in control groups and MLT-treated groups in BALB/c mice.** Photomicrographs of histological examination of liver, kidney, spleen, thymus, bone marrow, mesenteric lymph node, and Peyer’s lymph node (**a**–**g**) (100×) in BALB/c mice in the negative control group, the low-dose group, the medium-dose group, the high-dose group, and the positive control group.

Histological examinations were performed and pathological changes were observed, such as atrophy of splenic white pulp in the low-dose group (1/10); hepatocellular necrosis in a small lesion with a few inflammatory cells in filtrates in the medium-dose group (1/10); edema of hepatocytes (8/10) and mild atrophy in the thymus in the high-dose group (1/10). In the positive control group, more pathology changes were found, including: moderate splenic atrophy (3/10), reduction or disappearance of germinal center in lymphonodulus, mild splenic atrophy (7/10), mild thymic atrophy (7/10), and moderate thymic atrophy (3/10); lymphoid node atrophied, follicle structure vanished or unclear, lymphocyte reduction, and fibrous tissue hyperplasia (10/10); Peyer’s patches atrophy, follicle structure unclear, and reduction or disappearance of germinal center (10/10); decrease in the number of bone marrow stem cells and adipose hyperplasia (10/10) (Figure 2). The splenic germinal center in the medium-dose group was significantly greater than that in the negative control group (*p* < 0.05); however, there was no dose–response relationship (Table S5). Tissues and organs not mentioned above had no visible pathological changes.

### 3.6. Humoral Immunity

#### 3.6.1. PFC and HC_50_

**Figure 3:**
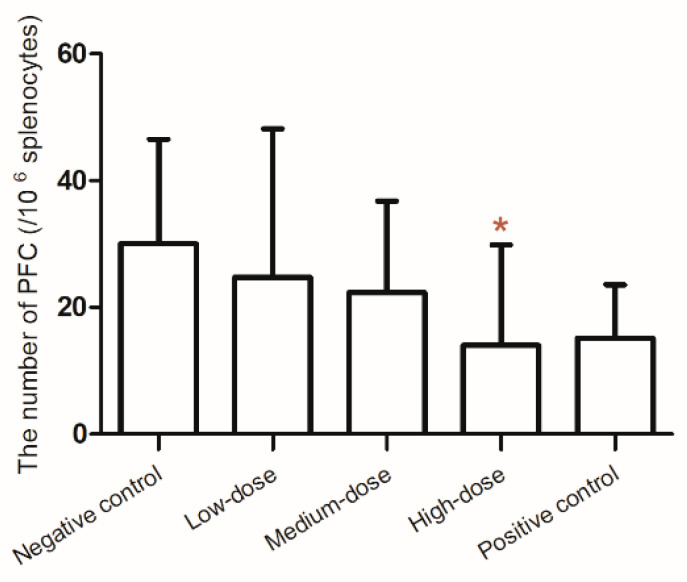
Effects on the numbers of plaque-forming cells (PFC) after exposure to MLT. Note: * statistically significant difference from the negative control group at *p* < 0.05. The PFC number per 10^6^ splenocytes in the high-dose group was significantly lower than that in the negative control group.

The number of PFCs decreased with increasing MLT dose and was significantly lower in the high-dose group than in the negative control group (Figure 3). Specifically, the PFC count per 10^6^ splenocytes in the high-dose group was approximately 50% lower than in the negative control group (*p* < 0.05), indicating marked suppression of the humoral immune response at this exposure level. No significant change in HC_50_ was observed (Table 3).

#### 3.6.2. Serum Immunoglobulin and Cytokines

**Figure 4:**
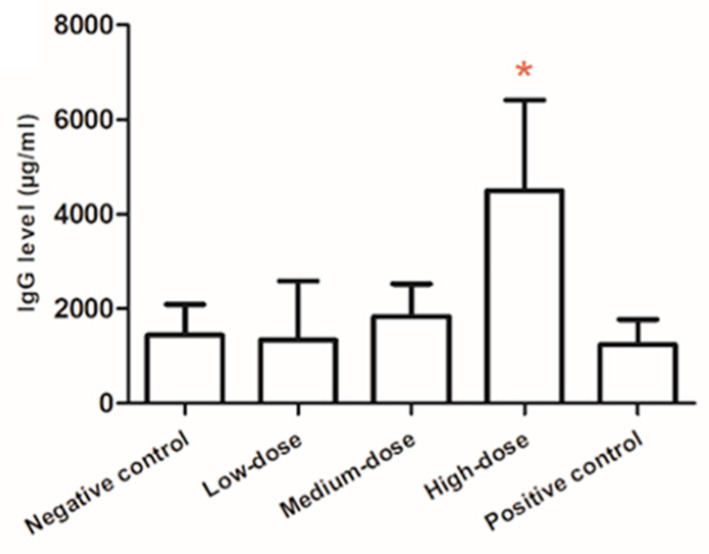
The serum IgG levels in the control groups and MLT-exposed groups. Note: * statistically significant difference from the negative control group, the low-dose group, the medium-dose group, and the positive control group at *p* < 0.05. The serum IgG level was abnormally elevated in the high-dose group.

IgG level in the high-dose group (4503.0 ± 1915.0 μg/mL) was significantly higher than that in the negative control (1440.0 ± 657.0 μg/mL), low-dose (1340.5 ± 1251.5 μg/mL), and middle-dose groups (1840.5 ± 688.0 μg/mL), and there was a clear dose–response relationship. The serum IgA level in the high-dose group (11.84 ± 5.04 μg/mL) was significantly higher than that in the low-dose (5.68 ± 6.48 μg/mL) and middle-dose groups (4.96 ± 3.84 μg/mL) (*p* < 0.05), but did not differ from the negative control. No significant differences in IgM levels were observed among the groups (Table 3 and Figure 4). Notably, this marked increase in serum IgG at the high dose contrasts with the significant reduction in PFC count observed in the same dose group (Figure 3). While the PFC assay measures the number of antibody-producing cells at a single time point, serum IgG levels reflect cumulative antibody production and clearance over time. This apparent discrepancy may indicate enhanced per-cell antibody secretion, prolonged antibody half-life, or differential effects of MLT on early versus late stages of B-cell differentiation. As shown in Table 3, the IL-5 level in the middle-dose group was significantly lower than that in the negative control group; IL-10 levels in the low-dose and the medium-dose groups were significantly lower than that in the high-dose group, and IL-10 level in the low-dose group was significantly lower than that in the negative control group.

### 3.7. Cellular Immunity

The mitogen-induced splenic lymphocyte proliferation, CTL cell activity, and DTH reaction were used to detect cellular immunity. Compared with the negative control group, CY inhibited the mitogen-induced splenic lymphocyte proliferation and the reaction of DTH. In contrast, none of these cellular immunity parameters were significantly altered in any MLT-treated group, including the high-dose group (Table 3). This lack of effect was notable given the significant changes observed in humoral immune endpoints such as PFC reduction and elevated IgG levels, indicating that MLT exposure selectively affected humoral immunity while leaving cellular immune responses largely intact under the conditions of this study.

### 3.8. Non-Specific Immunity

No significant changes were observed in either the carbon clearance test or the NK cell activity test (Table 3). Together with the absence of effects on cellular immune parameters, these findings indicated that MLT exposure in this study selectively affected humoral immune responses, particularly PFC counts and immunoglobulin levels, while leaving nonspecific immune functions and cell-mediated immunity largely unaffected. This pattern of selective immunomodulation suggested that MLT may target specific components of the adaptive humoral immune system without broadly compromising innate or cellular immune defenses under the current exposure conditions.

## 4. Discussion

In this study, immunotoxicity tests on BALB/c mice exposed to MLT at doses of 16, 65, and 258 mg/kg bw revealed selective effects on both immunopathological and humoral immune parameters. Notably, even the lowest dose (16 mg/kg bw) induced significant changes, including increased thymus weight and reduced levels of IL-10, NEUT, polychromatic erythroblasts, and monocytes, as well as atrophy of the splenic white pulp. In contrast, a dose-dependent elevation in serum IgG was observed, further supporting the immunomodulatory potential of MLT. These findings indicated that MLT exerts significant immunotoxic effects, with the LOAEL preliminarily identified as 16 mg/kg bw based on the alterations observed at this dose.

The mice in the positive control group were treated with CY to establish an immunosuppressed model. Compared with the negative control group, CY significantly decreased the spleen weight, WBC value, LYM value, MON value, NRUT value, %B cells, PFC/10^6^ splenocytes, mitogen-induced splenic lymphocyte proliferation, and the reaction of DTH, and significantly increased the GLU value, %T cells, %Th cells, and Th/Ts ratio. In addition, histopathology changes in the spleen, thymus, lymph nodes, and Peyer’s lymph nodes were also observed. These results were consistent with previous studies on the CY-induced immunosuppressive model [27,28]. It can be concluded that the immunosuppressed animal model was successfully established as the positive control in this study.

The WBC count with differential measured the number of leukocytes and the percentages of each type, including NEUT, BAS, EOS, monocytes, and lymphocytes, and provided information about the immune system [29]. As MLT concentration increased, leukocyte levels decreased, showing an obvious dose–response relationship in this study. Both the count and the percentage of BAS in the high-dose group were significantly lower than those in the other two MLT-treated groups. BAS were increasingly recognized as important regulators of immune responses, particularly in the context of Th2-type inflammation, allergic reactions, and immune modulation [30,31,32]. They may contribute to the initiation and amplification of adaptive immune responses through the release of cytokines such as IL-4 and IL-13, which promoted B-cell differentiation and immunoglobulin class switching [30,31,32]. The significant reduction in BAS levels observed at the high dose may therefore reflect a disruption of this regulatory axis, potentially contributing to the altered immunoglobulin profiles (e.g., elevated IgG) and suppressed PFC responses observed in the same dose group. Alternatively, basopenia could indicate bone marrow suppression or increased margination of these cells into tissues, consistent with the broader hematopoietic alterations noted in this study (e.g., reduced polychromatic erythroblasts). Furthermore, as a major effector in both the inherent and adaptive immunity of mammals, the NEUT played a particularly important role in complex immune regulation [33]. In general, the concentration of serum NEUT was in a steady state in the body, and the NEUT level in the blood will decrease when NEUTs enter tissues where inflammation occurred or when bone marrow cells were functionally impaired [34]. The reduction in NEUT levels in the low- and middle-dose groups suggested that a relatively lower dose of MLT negatively impacted NEUT homeostasis; in turn, the risk of infection might also be dramatically increased.

The PFC assay is widely regarded as the “gold standard” for immunotoxicity testing due to its high sensitivity and predictive value. Studies have shown that the PFC assay and immunophenotyping assays were concordant 91% of the time, and both were considered the most sensitive measures for detecting immunotoxic effects [20,35]. The present results showed that high-dose MLT (258 mg/kg bw) significantly reduced PFC counts compared with the negative control group, whereas no significant reductions were observed at the low (16 mg/kg bw) or middle (65 mg/kg bw) doses. This pattern indicated a threshold effect, with immunotoxicity manifesting only at the highest dose tested under the current exposure conditions. Although the PFC results for the low and middle doses were not significant, they still showed a downward trend. The identification of a significant PFC reduction specifically at 258 mg/kg bw supported the determination of a LOAEL for this endpoint at this dose. However, considering that other immunopathological and hematological alterations (e.g., thymus weight changes, IL-10 reduction, NEUT and monocyte decreases) were already evident at 16 mg/kg bw, the overall LOAEL for MLT immunotoxicity in this study remains 16 mg/kg bw based on the most sensitive endpoints. This highlighted the importance of integrating multiple immune parameters when establishing immunotoxic thresholds, as different endpoints may exhibit varying dose–response characteristics. The present results showed that high-dose MLT increased the percentage of B lymphocytes (CD^3−^CD^19+^), while the LYM count and the proportion of T lymphocytes (CD^3+^CD^19−^) in the same group had no significant change. It may indicate that the MLT (256 mg/kg bw) has the potential to disrupt lymphocyte homeostasis in BALB/c mice, and that B lymphocytes were much more sensitive to MLT toxicity than T lymphocytes.

ALT has been widely used as a liver injury biomarker to assess hepatic injury in experimental animals and to aid in the clinical diagnosis of patients [36]. According to the clinical chemistry parameters in the present study, the ALT level in the high-dose group was significantly increased while the levels of ALB and TP were reduced. In the meantime, hepatocyte necrosis in a small lesion with a small inflammatory cell infiltration in the middle-dose group, as well as edema of hepatocytes in the high-dose group, were observed. Similar results had been reported in two cases of drug-induced hepatic injury [37] and some hepatotoxicity studies of MLT, including histopathological and hematological changes [38,39,40]. In addition to that, the absolute and relative weights of the liver in the high-dose group increased, which might be induced by the metabolism of MLT. All the evidence listed above indicated that MLT (258 mg/kg bw) can induce liver injury in mice.

Except for the hepatic damage that had been discussed before, atrophy in the white pulp of the spleen (1/10) in the low-dose group and mild atrophy of the thymus in the high-dose group (1/10) were also causes for concern. Similar results were reported in the study of Ojha et al.: both acute and chronic exposure (LD_50_ and 0.5 LD_50_, respectively) of MLT caused splenic pathological changes [41]. Furthermore, their study clearly demonstrated that the DNA damage index of the spleen in the MLT-treated group was statistically significant when compared with the control group. The results of other studies supported the adverse effect induced by MLT on the thymocytes in vitro. Investigators exposed murine thymocytes to MLT and found a concentration-dependent increase of apoptosis after 12 h incubation [42]. Further research, carried out by Olgun et al., later revealed that oxidative stress induced by MLT played a key role in athymocyte apoptosis [42]. The mild atrophy of the thymus was not consistent with the results of significant increases in relative and absolute weights of the thymus in the medium- and high-dose groups. Still, it might be associated with increased thymus connective tissue.

Either PFC assay or ELISA were recommended by USEPA to assess the potential immunotoxicity of chemicals [43]. In particular, as one of the most sensitive methods, PFC assay has been proved to have a tight association with immunotoxicity (78% concordance) [44]. Humoral immunity tests showed that, with the increase of MLT concentration, the number of plaques per 10^6^ splenocytes decreased in a dose-dependent manner, and the maximum inhibition was shown in the high-dose group. Considering the mild splenic atrophy (1/10) in the high-dose group, the observed linear dose–response relationship indicated that MLT-induced splenic toxicity increased with increasing concentration. A similar trend was found in a 30-day study [45], which demonstrated that the higher dose of MLT will suppress the humoral immunity response. However, the serum IgG level was abnormally elevated in the high-dose group, which was inconsistent with the conclusion of PFC assay. A similar trend in serum IgG levels was found only in one study of workers in Poland, as early as 1985, who were occupationally exposed to organophosphorus pesticides, which showed an elevation in serum IgG levels compared with nonexposed participants [46]. This apparent discrepancy between reduced PFC and elevated serum IgG was particularly noteworthy and may reflect several underlying biological mechanisms. First, while the PFC assay measured the number of antibody-producing cells in the spleen at a single time point, serum IgG levels represent cumulative antibody production and clearance over time; thus, enhanced antibody secretion per plasma cell or a prolonged IgG half-life could explain elevated serum levels despite fewer PFC. Second, MLT exposure may differentially affect B-cell subpopulations or induce isotype switching toward IgG production, consistent with the observed increase in B-lymphocyte percentages (CD^3−^CD^19+^). Third, the elevated IgG could reflect a compensatory immune activation or a shift toward a Th2-type response, as suggested by the altered IL-10 observed in this study. Finally, the reduction in regulatory components such as IL-10 at lower doses may contribute to dysregulated humoral immunity. The possible reasons for the dramatic difference in the distribution of IgG in the serum and spleen might be as follows: a) the impaired function of the spleen resulted in a decrease in the level of IgG synthesized in the lymph nodes of the spleen, but the function of lymph nodes in other tissues was normal; b) the repeated high dose of MLT as an antigen stimulated the plasma cells (effector B cells) to secrete more IgG; c) intestinal metabolizing toxicants of MLT might cause IgG elevation; d) other mechanisms of immune dysfunction. Therefore, its potential mechanism needed further exploration. In addition, the non-monotonic dose response observed for total IgA levels aligned with the growing recognition of complex dose–response relationships in immunotoxicology. Potential mechanisms included differential receptor activation [24], gut microbiota modulation [47], and oxidative stress [48]. Similar non-linear responses have been reported for other immunological endpoints following pesticide exposure [49,50], suggesting that immune parameters may be particularly susceptible to biphasic modulation by xenobiotics. These findings highlighted the need for comprehensive dose–response assessments in immunotoxicity studies and the limitations of assuming monotonicity in risk extrapolation.

Furthermore, IL-10 levels in the low-dose and medium-dose groups were significantly decreased, and IL-5 levels in the medium-dose group were much lower than those in the negative control group. The cytokine IL-10 was mainly produced by T lymphocytes, B lymphocytes, macrophages, granulocytes, and dendritic cells, etc. [51], and was a recognized inflammatory and immunosuppressive factor involved in inflammatory response and immune reaction. Therefore, the reduction in IL-10 levels in the low- and middle-dose groups was considered to be partly related to the decrease in NEUTs, which made up the majority of granulocytes and were responsible for secreting IL-10. In addition, exposure to MLT did not affect the phagocytic function of splenic macrophages, mitogen-induced splenic lymphocyte proliferation, cytotoxic T-lymphocyte response, serum half-life hemolysis value, the increase of footpad thickness, and NK cell activity, which indicates that the cellular immune functions and non-specific immune functions in BALB/c mice might not be sensitive to MLT.

At present, the immunotoxicity mechanism of MLT is not fully understood. According to existing research, MLT can cause immunotoxicity through non-cholinergic mechanisms. Its metabolites can directly interact with cytoskeletal proteins (such as actin and microtubule proteins) and inhibit their aggregation kinetics, and activate Rho GTPase family members Ras-related C3 botulinum toxin substrate 1 and cell division control protein 42 to drive skeletal reorganization, pseudopodia formation, and abnormal migration of immune cells such as macrophages, thereby interfering with their chemotaxis, phagocytosis, and immune surveillance functions [24,52]. Meanwhile, long-term low-dose exposure can affect the proportions of T lymphocyte subsets. It may indirectly inhibit humoral and cellular immune responses by inducing oxidative stress and neuroendocrine interference, ultimately resulting in immunosuppression or autoimmune tendencies [50].

Our study had several advantages, including a relatively complete immunotoxicity test for MLT. It found that MLT can disrupt immune homeostasis in BALB/c mice at relatively low doses, with significant adverse effects on humoral immune parameters and pathological damage to immune organs, such as the spleen. Based on this, the LOAEL of MLT on the immune system was 16 mg/kg bw. At this dose, significant alterations were observed in multiple parameters: increased thymus weight, reduced IL-10 levels, decreased NEUT and monocyte counts, reduced polychromatic erythroblasts, and splenic white pulp atrophy. However, these effects were not consistently observed across all related endpoints, and some lacked clear dose–response relationships. Therefore, while 16 mg/kg bw may represent a LOAEL based on the most sensitive endpoints, this conclusion should be considered preliminary. Confirmatory studies with additional dose levels below 16 mg/kg bw were needed to establish a definitive NOAEL. Based on this, the LOAEL of MLT on the immune system was considered to be 16 mg/kg bw, with the understanding that this represented a conservative estimate derived from the lowest dose tested. To derive an acceptable daily intake (ADI) from this LOAEL, we applied a safety factor of 1000. This composite factor accounts for: (1) extrapolation from a LOAEL to a NOAEL (typically a factor of 10), as the present study identified effects at the lowest dose tested without a clear NOAEL; (2) interspecies differences between mice and humans (factor of 10); and (3) intraspecies variability in human sensitivity (factor of 10). The calculation was as follows: ADI = LOAEL (16 mg/kg bw)/1000 = 0.016 mg/kg bw [53]. According to current research, the ADI for MLT is 0.016 mg/kg bw. This is far lower than the current regulation of 0.3 mg/kg bw, which was specified by the Joint WHO/FAO Meeting on Pesticide Residues in 1997 [54] and reaffirmed in 2016 [55]. This conclusion was based on an unobserved adverse-effect level of 29 mg/kg bw in a chronic toxicity and carcinogenicity study in rats, using a safety factor of 100. While the present study focused exclusively on immunological endpoints and did not directly compare immunotoxicity with other toxicity outcomes within the same experimental framework, the substantially lower ADI derived from immunotoxicity data when compared with the regulatory ADI was based on traditional toxicity studies. It suggested that immunotoxicity may be a more sensitive endpoint for MLT exposure. This inference was drawn from cross-study comparisons and should be interpreted with appropriate caution. Nonetheless, it highlighted the potential importance of incorporating immunotoxicity assessments into chemical safety evaluations, as standard toxicological studies may not capture immune system adverse effects that manifest at lower doses [56,57]. This indicated that the immune system was more sensitive to MLT than other toxicity endpoints. Therefore, it was necessary to incorporate an immunotoxicity assessment into pesticide registration management and risk assessment of the daily diet. However, this study still had limitations, mainly reflected in the lack of further exploration of the immunotoxicity mechanism of MLT. Although some previous studies had explored its potential mechanisms, the relevant content was insufficient, and there was no in-depth study of humoral immunity and pathological damage to immune organs. Furthermore, several immunological parameters, including cytokine levels (e.g., IL-2, IL-4, IFN), immunoglobulin concentrations (e.g., IgA), and lymphocyte subpopulations did not exhibit clear dose–response trends across the tested dose range. This lack of consistency raised questions regarding the robustness of some findings. It may reflect inherent biological variability in immune responses, experimental variation, or insufficient statistical power due to the sample size (*n* = 10 per group). It is also possible that certain immune endpoints followed non-monotonic dose–response patterns, as observed for IL-5 and IL-10 in this study, which could complicate interpretation. These inconsistencies highlighted the need for confirmatory studies with larger sample sizes and additional intermediate dose levels to better characterize dose–response relationships and distinguish true biological effects from experimental noise. While the doses used in this study (16–258 mg/kg bw) were higher than typical human exposure levels or environmentally relevant concentrations, they were selected based on acute toxicity profile to establish a dose–response relationship and identify potential immunotoxic effects. These findings provided a foundation for understanding the hazards associated with MLT exposure and underscore the need for further studies using lower, more environmentally relevant doses to better assess real-world risks.

## 5. Conclusions

Given the toxicological evaluation results and the need for pesticide safety management, the immunotoxicity of MLT should serve as an important basis for relevant safety standards, providing a reference for improving pesticide registration management and immunotoxicity evaluation guidelines. Nonetheless, the proposed LOAEL should be interpreted with caution, given its preliminary nature and the absence of a clear NOAEL, underscoring the need for further studies with lower doses and mechanistic investigations to confirm these findings.

## Figures and Tables

**Table 1:** Effects on hematology parameters in mice after 30 days of feeding.

Group	Negative Control	Low-Dose	Middle-Dose	High-Dose	Positive Control
RBC (×10^12^ L^−1^)	10.67 ± 1.14	10.8 ± 1.14	10.59 ± 0.82	10.27 ± 1.48	10.70 ± 2.11
HGB (g L^−1^)	174.2 ± 16.6	172.6 ± 20.2	174.9 ± 12.7	171.1 ± 20.9	176.3 ± 37.3
HCT (%)	49.3 ± 5.2	49.2 ± 5.1	50.6 ± 3.6	48.1 ± 6.6	50.7 ± 9.9
MCV (fL)	46.2 ± 1.0	487.5 ± 1.1	47.8 ± 0.7	46.9 ± 1.1	47.5 ± 0.9
MCH (pg)	16.4 ± 0.4	16.6 ± 0.4	16.6 ± 0.7	16.7 ± 0.6	16.4 ± 0.4
MCHC (g L^−1^)	353.5 ± 6.8	350.2 ± 11.5	346.1 ± 13.7	356.1 ± 8.7	346.3 ± 10.8
RDW (%)	13.38 ± 0.31	13.15 ± 0.25	13.32 ± 0.36	13.69 ± 0.56	13.63 ± 0.39
PLT (×10^9^ L^−1^)	717.2 ± 92.8	806.7 ± 76.7 ^d^	758.4 ± 70.1	645.3 ± 158.1 ^b^	687.4 ± 105.8
PCT (%)	0.20 ± 0.03	0.22 ± 0.05	0.21 ± 0.04	0.18 ± 0.06	0.20 ± 0.05
MPV (fL)	2.77 ± 0.35	2.75 ± 0.43	2.73 ± 0.31	2.76 ± 0.36	2.88 ± 0.36
PDW (%)	17.64 ± 0.75	17.45 ± 0.93	17.57 ± 0.89	17.82 ± 0.83	17.69 ± 0.85
WBC (×10^9^ L^−1^)	8.11 ± 2.47 ^d,e^	7.49 ± 1.39 ^e^	7.08 ± 2.08 ^e^	6.18 ± 2.29 ^a,e^	3.81 ± 1.98 ^a,b,c,d^
LYM (×10^9^ L^−1^)	4.89 ± 1.29 ^e^	4.34 ± 2.09 ^e^	5.35 ± 1.59 ^e^	4.65 ± 1.40 ^e^	2.41 ± 1.81 ^a,b,c,d^
(%)	62.46 ± 15.64	67.20 ± 9.23	70.24 ± 10.08 ^e^	66.55 ± 12.43	58.02 ± 18.18 ^c^
MON (×10^9^ L^−1^)	0.37 ± 0.24 ^e^	0.39 ± 0.31 ^e^	0.42 ± 0.24 ^e^	0.38 ± 0.19 ^e^	0.08 ± 0.12 ^a,b,c,d^
(%)	5.39 ± 4.46	5.35 ± 3.57	5.40 ± 3.07	5.87 ± 3.78	2.98 ± 4.51
NEUT (×10^9^ L^−1^)	2.82 ± 2.21 ^b,c,e^	1.41 ± 0.35 ^a^	1.66 ± 0.66 ^a^	2.05 ± 1.36	1.32 ± 0.59 ^a^
(%)	31.36 ± 18.93 ^d^	26.60 ± 12.56	23.44 ± 10.92 ^e^	27.18 ± 15.12	38.28 ± 19.15 ^c^
EOS (×10^9^ L^−1^)	0.01 ± 0.03	0.00 ± 0.00	0.00 ± 0.00	0.00 ± 0.00	0.00 ± 0.00
(%)	0.40 ± 0.58	0.26 ± 0.11	0.23 ± 0.10	0.15 ± 0.13	0.42 ± 0.16
BAS (×10^9^ L^−1^)	0.02 ± 0.04 ^c^	0.04 ± 0.05 ^d^	0.06 ± 0.07 ^a,d^	0.00 ± 0.00 ^b,c^	0.00 ± 0.00
(%)	0.39 ± 0.34 ^c^	0.59 ± 0.34 ^d,e^	0.69 ± 0.58 ^a,d,e^	0.25 ± 0.14 ^b,c^	0.30 ± 0.40 ^b,c^

Note: Data were given as mean ± SD (*n* = 10). ^a^: statistically significant difference from the negative control group at *p* < 0.05; ^b^: statistically significant difference from the low-dose group at *p* < 0.05; ^c^: statistically significant difference from the middle-dose group at *p* < 0.05; ^d^: statistically significant difference from the high-dose group at *p* < 0.05; ^e^: statistically significant difference from the positive control group at *p* < 0.05.

**Table 2:** Effects on clinical chemistry parameters in mice after 30 days of feeding.

Group	Negative Control	Low-Dose Group	Middle-Dose Group	High-Dose Group	Positive Control
ALT (U L^−1^)	28.4 ± 4.2	22.3 ± 2.0 ^d,e^	22.0 ± 1.0 ^d,e^	31.9 ± 11.8 ^b,c^	33.7 ± 10.7 ^b,c^
AST (U L^−1^)	151.7 ± 35.4	137.9 ± 24.1 ^e^	128.8 ± 20.0 ^e^	150.0 ± 32.6	165.4 ± 31.2 ^b,c^
TP (g L^−1^)	63.45 ± 3.81 ^d^	65.21 ± 3.59 ^c,d^	59.86 ± 5.04 ^b,e^	56.95 ± 3.26 ^a,b,e^	65.62 ± 3.49 ^c,d^
ALB (g L^−1^)	39.38 ± 1.74 ^d^	40.22 ± 0.80 ^c,d,e^	38.45 ± 1.12 ^b,d^	36.58 ± 2.35 ^a,b,c,e^	38.33 ± 1.67 ^b,d^
ALP (U L^−1^)	114.5 ± 6.6	122.4 ± 14.4	110.7 ± 20.3	111.8 ± 18.0	114.6 ± 10.8
GLU (mmol L^−1^)	1.97 ± 0.90 ^c,e^	2.54 ± 1.23	3.52 ± 0.84 ^a,d^	1.85 ± 1.57 ^c,e^	2.60 ± 1.03 ^a,d^
BUN (mmol L^−1^)	7.67 ± 0.75	7.32 ± 0.92	8.00 ± 0.86	7.33 ± 1.56	8.78 ± 1.13
CRE (μmol L^−1^)	49.66 ± 3.00	49.37 ± 4.55	48.49 ± 5.12	49.29 ± 9.61	56.47 ± 15.69
CHO (mmol L^−1^)	2.08 ± 0.20 ^d^	2.14 ± 0.26 ^d^	2.21 ± 0.31	2.46 ± 0.30 ^a,b^	2.20 ± 0.24
TG (mmol L^−1^)	0.97 ± 0.21	0.98 ± 0.17	0.96 ± 0.13	1.02 ± 0.25	1.14 ± 0.20
Na (mmol L^−1^)	130.5 ± 8.2	130.4 ± 8.8	133.1 ± 6.2	132.8 ± 7.6	135.4 ± 16.5
K (mmol L^−1^)	6.5 ± 0.8	6.6 ± 0.8	6.4 ± 0.6	6.6 ± 0.3	6.5 ± 0.3
CA (mmol L^−1^)	1.4 ± 0.1	1.4 ± 0.1	1.4 ± 0.1	1.4 ± 0.0	1.4 ± 0.1

Note: Data were given as mean ± SD (*n* = 10). ^a^: statistically significant difference from the negative control group at *p* < 0.05; ^b^: statistically significant difference from the low-dose group at *p* < 0.05; ^c^: statistically significant difference from the middle-dose group at *p* < 0.05; ^d^: statistically significant difference from the high-dose group at *p* < 0.05; ^e^: statistically significant difference from the positive control group at *p* < 0.05.

**Table 3:** Effects on the number of splenocytes and parameters of humoral immunity, cellular immunity, and non-specific immunity in mice under MLT exposure.

Group	Negative Control	Low-Dose Group	Middle-Dose Group	High-Dose Group	Positive Control
**Basic Immune Organ Parameters**					
Number of splenocytes (10^8^/g spleen)	7.09 ± 2.01 ^e^	9.76 ± 2.81 ^e^	8.24 ± 1.81 ^e^	8.10 ± 2.06 ^e^	1.89 ± 1.38 ^a,b,c,d^
**Humoral Immunity**					
PFC (/10^6^ splenocytes)	30.0 ± 16.5 ^d,e^	24.8 ± 23.4	22.4 ± 14.4	14.0 ± 15.9 ^a^	15.2 ± 8.4 ^a^
HC_50_	276.2 ± 74.8	260.2 ± 56.3	256.2 ± 71.3	242.7 ± 39.6	227.8 ± 48.8
IgA (μg/mL)	8.64 ± 6.52	5.68 ± 6.48 ^d^	4.96 ± 3.84 ^d^	11.84 ± 5.04 ^b,c,e^	0.00 ± 0.00 ^d^
IgG (μg/mL)	1440.0 ± 657.0 ^d^	1340.5 ± 1251.5 ^d^	1840.5 ± 688.0 ^d^	4503.0 ± 1915.0 ^a,b,c,e^	1246.4 ± 533.0 ^d^
IgM (μg/mL)	92.4 ± 61.6	97.6 ± 39.2	114.4 ± 38.6	147.6 ± 70.0	124.3 ± 51.8
**Cytokine**					
IL-2 (pg/mL)	2.90 ± 0.89	2.74 ± 0.36	2.57 ± 1.06	3.10 ± 0.58	2.63 ± 0.98
IL-4 (pg/mL)	2.16 ± 0.31	2.07 ± 0.42	2.25 ± 0.43	2.43 ± 0.31	2.52 ± 0.26
IL-5 (pg/mL)	15.91 ± 6.10 ^c^	13.66 ± 5.93	10.75 ± 3.58 ^a^	13.41 ± 6.90	14.69 ± 4.09
IL-10 (pg/mL)	11.85 ± 5.43 ^b,c,e^	6.28 ± 5.11 ^a,d^	7.94 ± 3.87 ^a,d^	12.50 ± 9.25 ^b,c,e^	7.88 ± 3.27 ^a,d^
IFN (pg/mL)	6.49 ± 3.87	5.38 ± 2.03	6.06 ± 3.73	7.38 ± 5.05	4.27 ± 1.28
TNF (pg/mL)	7.50 ± 1.26	8.40 ± 4.12	8.41 ± 3.54	7.91 ± 2.32	8.16 ± 1.59
**Cellular Immunity**					
ConA-induced splenocyte proliferation	0.61 ± 0.22 ^e^	0.66 ± 0.10 ^e^	0.79 ± 0.22 ^e^	0.81 ± 0.20 ^e^	0.21 ± 0.16 ^a,b,c,d^
LPS-induced splenocyte proliferation	0.40 ± 0.18 ^e^	0.32 ± 0.11 ^e^	0.41 ± 0.16 ^e^	0.36 ± 0.17 ^e^	0.02 ± 0.10 ^a,b,c,d^
CTL cell activity	3.48 ± 3.54	6.46 ± 9.80	7.72 ± 17.40	2.76 ± 7.12	4.74 ± 5.53
Increase in footpad thickness (mm)	0.058 ± 0.043 ^e^	0.069 ± 0.034 ^e^	0.043 ± 0.037	0.074 ± 0.054 ^e^	0.019 ± 0.033 ^a,b,d^
**Non-specific Immunity**					
NK cell activity	14.06 ± 16.18	8.61 ± 9.86	4.15 ± 8.44	12.63 ± 5.87	13.08 ± 36.40
Macrophage phagocytosis	6.41 ± 1.60	5.73 ± 1.02	6.40 ± 1.11	5.67 ± 1.76	5.62 ± 4.43

Note: Data were given as mean ± SD (*n* = 10). ^a^: statistically significant difference from the negative control group at *p* < 0.05; ^b^: statistically significant difference from the low-dose group at *p* < 0.05; ^c^: statistically significant difference from the middle-dose group at *p* < 0.05; ^d^: statistically significant difference from the high-dose group at *p* < 0.05; ^e^: statistically significant difference from the positive control group at *p* < 0.05.

## Data Availability

The original contributions presented in this study are included in the article/Supplementary Materials. Further inquiries can be directed to the corresponding authors.

## Supplementary Materials

The following supporting information can be downloaded at: https://www.mdpi.com/article/10.3390/toxics14040279/s1, Table S1. The body weights of MLT-exposed mice at different time points; Table S2. Effects on the number of Peyer’s lymph glands, and terminal body weight and organ weight in mice; Table S3. Effects on phenotypic analysis of peripheral blood lymphocytes in mice; Table S4. Effects on classification of bone marrow cells in MLT-treated BALB/c mice; Table S5. Effects on spleen and Peyer’s patches in BALB/c mice after 30 days of feeding; Figure S1. Body weights of BALB/c mice at different time points; Figure S2. Effects on T lymphocyte (CD^3+^CD^19−^) and B lymphocyte (CD^3−^CD^19+^) after exposure to MLT for 30 days.
