# Supplementary material for: Evaluation of Immunotoxicity Induced by Organophosphorus Pesticide Malathion
Source: Toxics. 2026 Mar 26;14(4):279. doi: 10.3390/toxics14040279 (PMC13119638; doi:10.3390/toxics14040279)
Supplement: Supplementary file 1 [file toxics-14-00279-s001.zip › toxics-4200528-supplementary.pdf]

Supplementary Material

Table S1. The body weights of MLT-exposed mice at different time points.

Table S2. Effects on the number of Peyer's lymph gland, terminal body weight and organ weight in mice.

Table S3. Effects on phenotypic analysis of peripheral blood lymphocytes in mice.

Table S4. Effects on classification of bone marrow cells in MLT-treated BALB/c mice.

Table S5. Effects on spleen and Peyer's patches in BALB/c mice after 30 days of feeding.

Figure S1. Body weights of BALB/c mice at different time points.

Figure S2. Effects on T lymphocyte ($CD^{3+}CD^{19-}$) and B lymphocyte ($CD^{3-}CD^{19+}$) after exposure to MLT for 30 days.

Table S1. The body weights of MLT exposed mice at different time points.

Group	Week 0	Week 1	Week 2	Week 3	Week 4
Negative Control	19.4±0.9	19.1±1.1	19.9±1.1	19.1±1.1	19.4±0.9
Low Dose Group	19.6±1.9	19.4±1.6	20.3±1.7	19.7±1.7	20.2±1.7
Middle Dose Group	19.5±0.9	19.6±1.0	20.2±0.9	20.0±1.2	19.7±1.3
High Dose Group	19.2±1.3	19.4±1.1	19.3±0.9	19.4±1.1	20.0±1.1
Positive Control	19.4±1.3	19.6±1.1	19.8±0.9	20.1±1.2	20.0±1.0

Note: Data are given as mean ± *SD* (n=10).

Table S2. Effects on the number of Peyer's lymph gland, terminal body weight and organ weight in mice

	Negative control	Low dose group	Middle dose group	High dose group	Positive control
Terminal body weight (g)	18.6±1.0	18.6±1.4[e]	19.2±1.4[e]	18.1±1.1	17.6±0.9[b, c]
Number of Peyer's Patches (	8.4±1.0	7.3±0.9	8.8±2.1	8.8±2.2	7.9±2.0
Liver					
Absolute weight（g）	0.736±0.061[d]	0.727±0.069[d]	0.764±0.056[d]	0.861±0.110[a, b, c, e]	0.708±0.427[d]
Relative weight（%）	3.978±0.390[d]	3.957±0.187[d]	3.994±0.376[d]	4.760±0.659[a, b, c, e]	4.036±0.215[c, d]
Kidney					
Absolute weight（g）	0.208±0.168	0.203±0.023	0.213±0.017	0.221±0.024	0.216±0.015
Relative weight（%）	1.201±0.064[d, e]	1.090±0.085[d, e]	1.109±0.070[d, e]	1.222±0.129[a, b, c]	1.228±0.074[a, b, c]
Spleen					
Absolute weight（g）	0.059±0.006[e]	0.064±0.010[e]	0.064±0.008[e]	0.056±0.007	0.034±0.006[a, b, c]
Relative weight（%）	0.319±0.029[e]	0.343±0.043[e]	0.336±0.048[e]	0.307±0.025[e]	0.191±0.031 [a, b, c, d]
Thymus					
Absolute weight（g）	0.026±0.005[b, c]	0.034±0.010[a, e]	0.035±0.009[a, e]	0.030±0.010	0.021±0.007 [b, c]
Relative weight（%）	0.141±0.027[b, c]	0.182±0.047[a]	0.181±0.045[a]	0.167±0.049	0.118±0.041
Lymph gland					
Absolute weight（g）	0.006±0.002	0.006±0.003	0.007±0.003[d]	0.005±0.002	0.027±0.012
Relative weight（%）	0.031±0.010	0.035±0.014	0.038±0.016	0.027±0.012	0.030±0.016

Note: Data are given as *mean* ± *SD* (n=10).[a]: statistically significant different from the negative control group at P<0.05; [b]: statistically significant different from the low dose group at P<0.05; [c]: statistically significant different from the middle dose group at P<0.05; [d]: statistically significant different from the high dose group at P<0.05; [e]: statistically significant different from the positive control group at P<0.05.

Table S3. Effects on phenotypic analysis of peripheral blood lymphocytes in mice.

Group	$CD^{3-}CD^{19+}$ (%)	$CD^{3+}CD^{19-}$ (%)	$CD^{3-}CD^{49+}$ (%)	$CD^{3+}CD^{4+}$ (%)	$CD^{3+}CD^{8+}$ (%)	CD^{4+}/CD^{8+}
Negative control	29.2±4.7$^{d, e}$	63.5±5.5^{e}	5.5±1.9	52.4±5.8^{e}	11.1±1.0^{c}	4.8±0.8^{e}
Low dose group	30.4±10.6^{e}	62.8±11.2^{e}	6.4±2.1	52.0±11.1^{e}	10.9±1.3	4.8±1.2^{e}
Middle dose group	36.1±9.2^{e}	56.8±10.0^{e}	7.4±3.2	47.3±10.3^{e}	9.5±1.3^{a}	5.1±1.4^{e}
High dose group	37.0±7.3$^{a, e}$	56.0±6.8^{e}	6.1±1.0	46.1±6.2^{e}	10.4±1.8	4.7±1.0^{e}
Positive control	7.5±8.4$^{a, b, c, d}$	84.6±7.9$^{a, b, c, d}$	5.4±2.9	74.2±8.8$^{a, b, c, d}$	10.4±1.9	7.5±2.1$^{a, b, c, d}$

Note: Data are given as mean ± *SD* (n=10). ^{a}: statistically significant different from the negative control group at P<0.05; b: statistically significant different from the low dose group at P<0.05; ^{c}: statistically significant different from the middle dose group at P<0.05; ^{d}: statistically significant different from the high dose group at P<0.05; ^{e}: statistically significant different from the positive control group at P<0.05.

Table S4. Effects on classification of bone marrow cells in MLT-treated BALB/c mice.

Group	Negative control	Low dose group	Middle dose group	High dose group	Positive control
Myeloid series					
Promyelocyte	0.9±0.9	0.7±0.7	1.5±1.5[e]	1.0±1.9	0.1±0.3[c]
Myelocyte	0.0±0.0	0.0±0.0	0.0±0.0	0.0±0.0	0.0±0.0
Metamyelocyte	0.2±0.6	0.1±0.3	0.1±0.3	0.2±0.4	0.0±0.0
Stab granulocyte	12.0±5.2[e]	11.0±3.6	12.7±4.5[e]	9.7±5.0	6.7±5.7[a, c]
Segmented granulocyte	21.2±6.7[e]	21.0±7.8[e]	24.6±5.9[e]	27.6±12.1[e]	48.2±8.3[a, b, c, d]
Erythrocytes series					
Basophilic erythroblast	2.0±2.5	1.2±1.5	1.0±0.9	1.0±0.8	1.1±1.4
Polychromatic erythroblast	6.0±4.2[b, c, d, e]	2.3±1.6[a]	2.4±1.5[a]	1.4±1.5[a]	1.2±1.5[a]
Orthochromatic erythroblast	6.7±4.0	7.9±4.5	6.9±3.6	7.0±2.4	7.2±3.7
Lymphocytic series	50.0±10.9[e]	55.1±6.6[e]	50.6±7.6[e]	52.6±13.1[e]	35.5±8.0[a, b, c, d]
Monocytic series	1.0±0.8[b, c, d, e]	0.1±0.3[a]	0.2±0.4[a]	0.5±0.7[a, e]	0.0±0.0
Other myeloid cell series	0.0±0.0	0.1±0.3	0.0±0.0	0.0±0.0	0.0±0.0

Note: Data are given as mean ± *SD* (*n*=10). [a]: statistically significant different from the negative control group at $P<0.05$; [b]: statistically significant different from the low dose group at $P<0.05$; [c]: statistically significant different from the middle dose group at $P<0.05$; [d]: statistically significant different from the high dose group at P<0.05; [e]: statistically significant different from the positive control group at $P<0.05$.

Table S5. Effects on spleen and Peyer's patches in BALB/c mice after 30 days of feeding

Group	Negative control	Low dose group	Middle dose group	High dose group	Positive control
Spleen					
Periarterial lymphatic sheath (μm)	470.46±58.00[e]	462.73±62.56[e]	486.12±73.08[e]	474.15±69.01[e]	292.85±61.46[a, b, c, d]
Germinal center (μm)	87.82±79.19[c]	95.78±40.47	129.17±41.78[a]	114.86±20.41	120.23±35.14
Peyer's patch					
Lymphoid follicle (μm)	473.28±80.13	493.18±69.98	504.61±119.99	473.21±86.22	383.32±45.53
Germinal center (μm)	286.59±85.80[e]	314.34±142.89[e]	281.13±157.24[e]	306.06±77.61[e]	221.08±57.34[a, b, c, d]

Note: Data are given as mean ± *SD* (*n*=10). [a]: statistically significant different from the negative control group at $P<0.05$; [b]: statistically significant different from the low dose group at $P<0.05$; [c]: statistically significant different from the middle dose group at $P<0.05$; [d]: statistically significant different from the high dose group at $P<0.05$; [e]: statistically significant different from the positive control group at $P<0.05$.

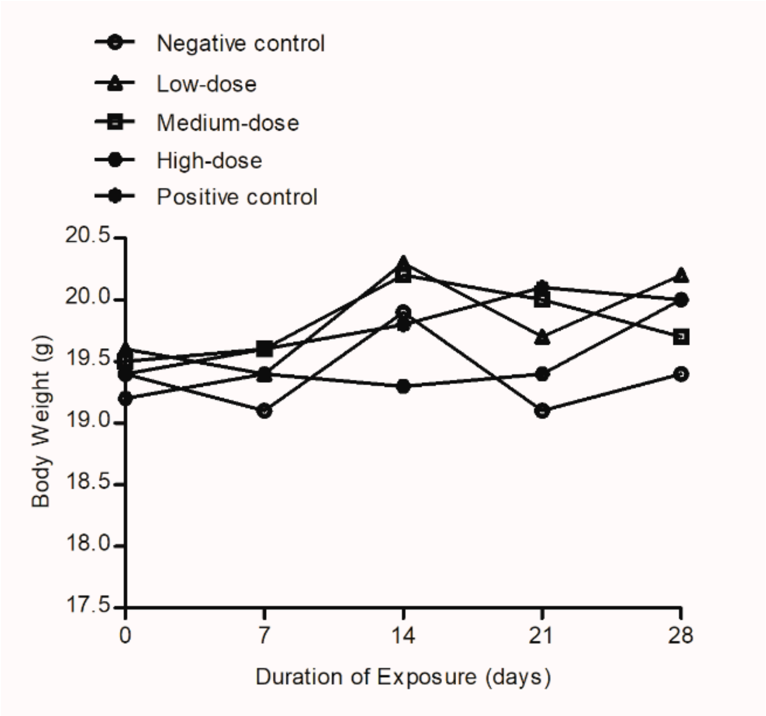

Figure S1. Body weights of BALB/c mice at different time points.

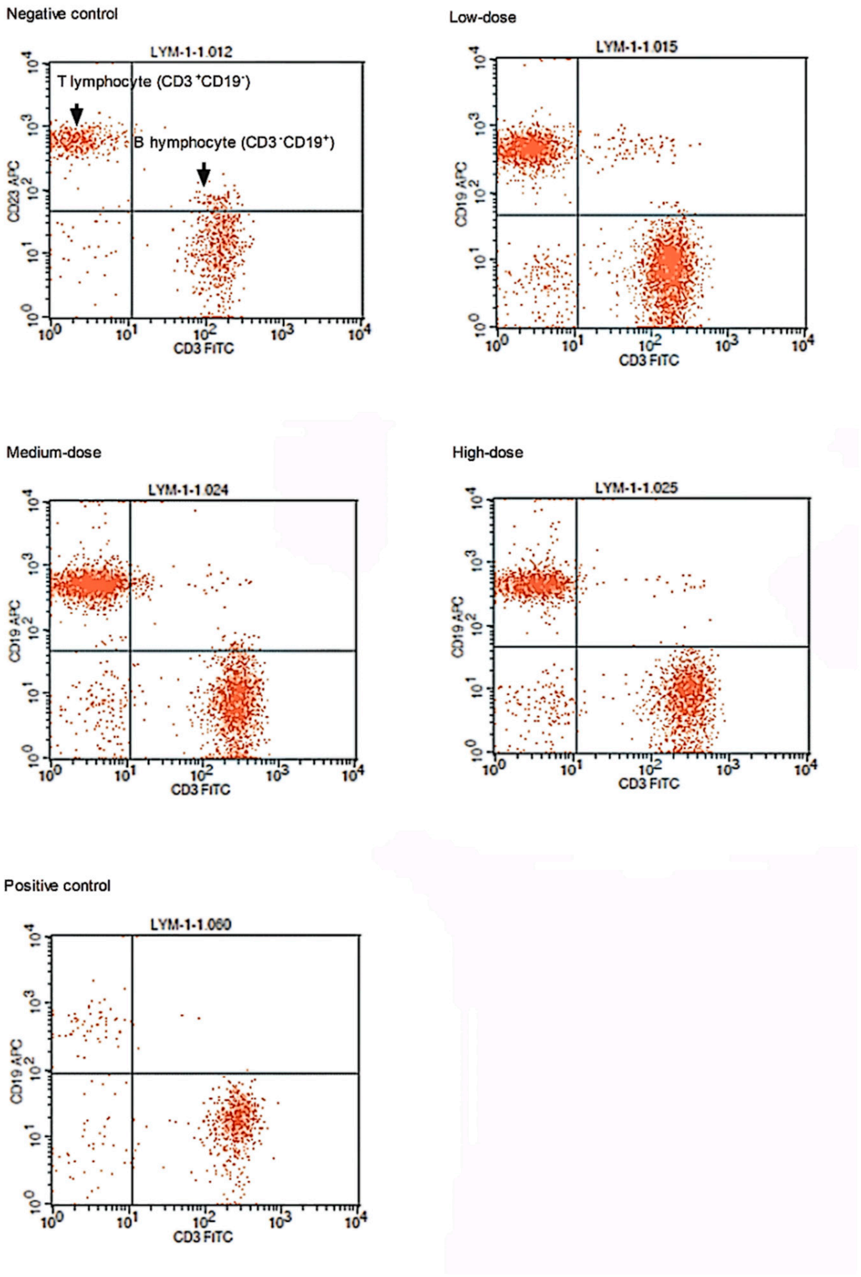

Figure S2. Effects on T lymphocyte ($CD^{3+}CD^{19-}$) and B lymphocyte ($CD^{3-}CD^{19+}$) after exposure to MLT for 30 days.
